# Knowledge, attitude, and practices among Iranian neurologists toward evidence-based medicine

**Published:** 2017-01-05

**Authors:** Kaveh Shafiei, Fatemeh Sedaghati

**Affiliations:** 1 Department of Neurology, School of Medicine, Kerman University of Medical Sciences, Kerman, Iran; 2 Neurology Research Centre, Kerman University of Medical Sciences, Kerman, Iran

**Keywords:** Evidence-Based Medicine, Knowledge, Attitude, Neurologist, Iran

## Abstract

**Background:** Evidence-based medicine (EBM) is a current practice in medicine to produce clinical practice guidelines from well-designed, randomized, controlled trials. We studied knowledge, attitude, and practice of EBM of neurologists who participated in the Iranian congress of neurology.

**Methods:** A self-administered anonymous questionnaire was distributed and filled by neurologists.

**Results:** A total of 200 neurologists were randomly sampled and with response rate of 56%. 33.9% of responder had previously participated in EBM courses. The average total knowledge score was 4.05 ± 0.80 out of a maximum possible score of 5.0. Textbooks were still the most favorite source of knowledge for our neurologists. A lack of time was the highest, and motivation the least mentioned barrier in using EBM.

**Conclusion:** Overall, the Iranian neurologist had acceptable knowledge and attitude toward EBM and had same similar as found in other studies.

## Introduction

As neurologists, we are living in an era of exponential growth of knowledge. Needless to say that practicing medicine is very different today than it was 15 years ago and even is going to be more different in 2020. This will be challenging, especially with the current rate of medical knowledge doubling every 3 years. The rate of doubling is expected to be every 73 days by 2020.^1^

The evidence-based medicine (EBM) is the process of producing clinical guidelines from well-designed, randomized and controlled trials, published in literature databases.^2^^,^^3^ Hence, the main goal of EBM is to optimize clinical decision-making and keep health practitioners’ knowledge up-to-date.^4^

The knowledge of medicine is growing very rapidly and using all available resources by medical professionals to be up-to-date is becoming more difficult (if not impossible). Therefore, the physician’s attitude toward EBM has increased enormously and positively.^5^

By learning how to practice EBM and adopting evidence-based practice protocols, medical professionals can put themselves in tandem of medical advances, and this can be helpful to enhance their clinical performances.^6^

Neurologists, like other medical professionals worldwide, are being encouraged to apply EBM to improve their clinical care.

To the best of our knowledge, little is known about knowledge, attitude, and practice of EBM among Iranian neurologists. 

To gain more information, we designed and conducted a survey.

The aim of our study was to answer five research questions about these topics:

- What are main resources for Iranian neurologist to find their clinical questions?
- Do Iranian neurologists know enough about important clinical aspects of EBM?
- How frequently they use EBM methods in their clinical practice?
- What are their limitations to use EBM?

Have they ever participated in EBM workshops?

## Materials and Methods

A cross-sectional observation study was conducted on Iranian neurologists who participated in the 22^nd ^Iranian Congress of Neurology and Electrophysiology, held in Tehran, in 2014. We chose this congress because it was popular and the participation level was expected to be high. 

We used convenience-sampling procedure to select participants, and a self-administered anonymous questionnaire was distributed to participants. 

The questioner had been translated, validated and used by Navabi, et al.^7^ The scientific reliability of the questionnaire was confirmed and labeled as optimal by previous studies, and all of the items were deemed highly appropriate to use for this group of physicians.

The questionnaire included 22 questions and evaluated background data, knowledge, attitude, and practice, in four sections. The first section covered background data, including gender, age, level of education, and type of practice.

The second section evaluated their knowledge level of EBM. They were asked to answer five statements with a three-point Likert scale: “correct,” “incorrect,” or “do not know.” The total knowledge score was calculated by scoring the answers: one point for correct answer and zero point for wrong or for “do not know” answers. The sum of the scores was the basis of calculating their level of knowledge. 

In the next section, neurologists were asked to state their most favorite sources for getting their medical information. They were also tested for their knowledge about EBM terms such as EBM, clinical effectiveness, relative risk, systematic review, critical appraisal, and Cochrane collaboration. They had to state their knowledge level by “know well,” “little is known,” or “do not know anything.”

The participants were given enough time, and the questionnaires were done anonymously. 

One of the objectives of the survey was to understand their level of online medical resources usage, and their limitations in using EBM. We were also interested to know if they had participated in other EBM workshops. 

Data were analyzed using Pearson’s chi-squared test for the comparison of frequencies and mean scores and the SPSS (version 16, SPSS Inc., Chicago, IL, USA). A P < 0.05 was considered significant.

## Results

A total of 200 survey questioners were distributed among neurologists who attended the congress, 113 of them returned the survey questioners, making 56% response rate. 81.4% of the responders were male with mean age of 44.4 years. 33.6% of them were university faculty members with 11.8 years of work experience after finishing their neurology training. 

The responders got the mean score of 4.05 ± 0.80 out of a maximum score of 5, regarding their knowledge.

Our survey responders mentioned their sources of acquiring professional knowledge. 84.1% of our responder used textbooks to get their clinical answers. Other sources of medical knowledge were online resources (79.6%), expert opinion (61.9%) and personal experiences (24.8%), respectively. 

**Figure 1:**
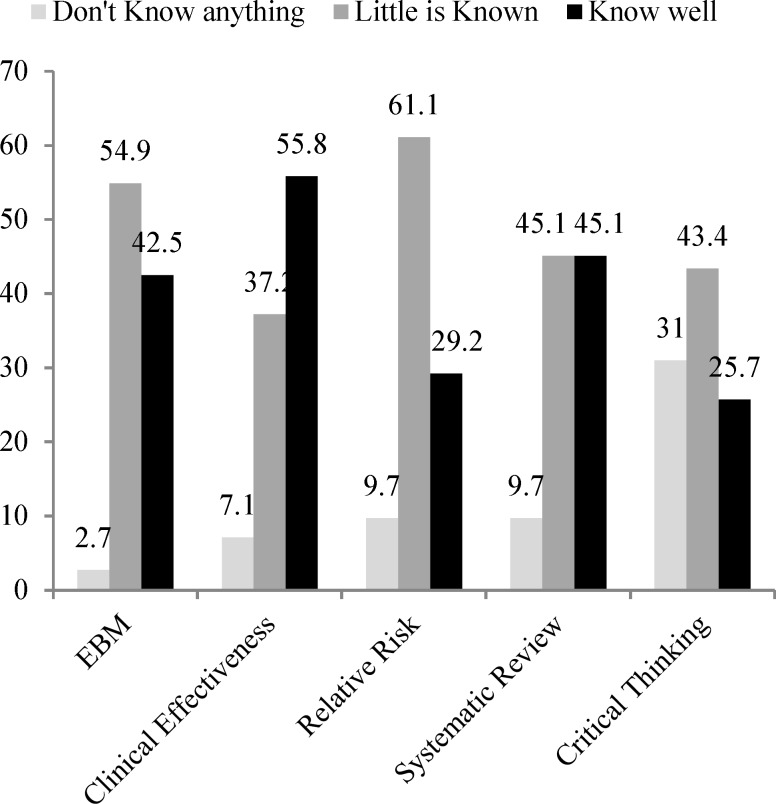
The statement of the Iranian neurologists regarding selected evidence-based terms. (n = 113)

Only 33.9% of the responders admitted that had attended EBM courses before. The “Clinical Effectiveness” was the most familiar and the “EBM” was least familiar term among the participants. More detail is shown in figure 1.

**Figure 2:**
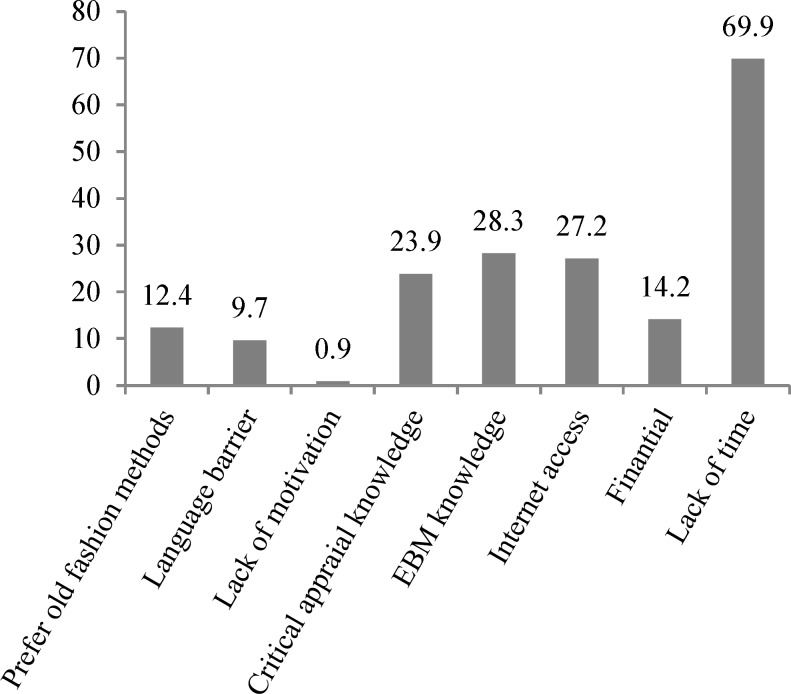
Frequency of mentioned barriers in using evidence-based medicine (EBM) by Iranian neurologists

We also asked about limitations in using EBM by Iranian neurologists. Figure 2 shows the frequencies of these barriers. By far the highest mentioned barrier was lack of time (69.9%) and on the other hand, motivation was the least barrier (0.9%). 

On average participants in our survey spent 1-5 hours/week online to find their clinical answers.

## Discussion

This was a questionnaire-based survey on the Iranian neurologist who participated in the annual congress of Iranian Neurology Associations. An acceptable numbers of them kindly returned the self-administered questioner.^8^

In general, neurologists who participated in our survey positively welcomed EBM and had acceptable basic knowledge and understanding of EBM. Compared to the same survey on dentists by Navabi, et al.,^7^ neurologists had better mean total knowledge score and were more likely to have participated in EBM workshop before. Both groups admitted that they usually prefer textbooks than other sources to resolve uncertainty in clinical practice. A lack of time was expressed as the predominant barrier to use EBM. 

Ghahremanfard, et al.^9^ studied knowledge and attitude toward EBM among medical students in Semnan, Iran in 2014. Based on their results, only 24.5% of medical students had good basic information and familiarity with the term of EBM. The positive attitude toward EBM existed in 89.3% of participants. Only 29% of medical students reported having had formal training in search strategies.

Our findings were quite similar to those of other studies conducted in the Middle East, which assessed the healthcare provider’s attitude toward EBM. They also showed that the major mentioned barriers to practicing EBM were lack of free personal time.^10^^,^^11^

Barghouti, et al.^8^ assessed Jordanian family practitioners’ attitudes toward and awareness of EBM in 2009. Only 20.4% of them had received formal training in research and critical appraisal. They also found that lack of personal time was the main perceived barrier to practicing EBM.

In a study on Australian general practitioners, the most commonly cited barrier to EBM was patient demand for treatment despite the lack of evidence for effectiveness. In those groups of physicians, the next most highly rated barriers were lack of time. They mentioned that lack of time was rated as a “very important barrier” by significantly more participants than lack of skills.^12^ Same findings have been reported in a Norwegian study in 2009.^13^

The evidence-based approach can be rationalized as the best treatment in resource-limited countries like Iran. It can also be the most cost-effective approach by reducing clinical practices that have no proven benefit. At present, EBM has major barriers, such as its inherent complexity, misperceptions, absence in medical curriculum, and unawareness of practicing clinicians.^14^

In general, physicians experience significant barriers to integrate EBM into clinical practice.^15^

These steps are recommended to overcome these barriers: effective teaching of skills of EBM during residency, motivating the established clinicians, formulating locally applicable guidelines, increasing the accessibility to internet, availing telemedicine facility at remote center and disseminating appropriate information via free journals or even newspapers. A strong political commitment is needed so that these steps can help to lay the foundation of EBM in Iran. 

## Conclusion

- Textbooks were main resources for Iranian neurologist to find their clinical questions. They less relied on expert opinion for that reason. We think, it is a major change in their view
- Iranian neurologists knew well enough about important clinical aspects and term in EBM practice
- They were using EBM methods in their clinical practice more than other medical practitioners in similar studies

They had good motivation on using EBM, but the lack of time was a major barrier in that way.

## Notes:

**How to cite this article:** Shafiei K, Sedaghati F. Knowledge, attitude, and practices among Iranian neurologists toward evidence-based medicine. Iran J Neurol 2017; 16(1): 26-9.
